# A system that delivers an antioxidant to mitochondria for the treatment of drug-induced liver injury

**DOI:** 10.1038/s41598-023-33893-7

**Published:** 2023-05-10

**Authors:** Mitsue Hibino, Masatoshi Maeki, Manabu Tokeshi, Yoichi Ishitsuka, Hideyoshi Harashima, Yuma Yamada

**Affiliations:** 1 Faculty of Pharmaceutical Sciences, Hokkaido University, Kita 12, Nishi 6, Kita-ku, Sapporo, 060-0812 Japan; 2 Faculty of Engineering, Hokkaido University, Sapporo, Japan; 3 Graduate School of Pharmaceutical Sciences, Kumamoto University, Kumamoto, Japan; 4 Japan Science and Technology Agency (JST) Fusion Oriented Research for Disruptive Science and Technology (FOREST) Program, Kawaguchi, Japan

**Keywords:** Drug delivery, Nanoparticles

## Abstract

Mitochondria, a major source of reactive oxygen species (ROS), are intimately involved in the response to oxidative stress in the body. The production of excessive ROS affects the balance between oxidative responses and antioxidant defense mechanisms thus perturbing mitochondrial function eventually leading to tissue injury. Therefore, antioxidant therapies that target mitochondria can be used to treat such diseases and improve general health. This study reports on an attempt to establish a system for delivering an antioxidant molecule coenzyme Q_10_ (CoQ_10_) to mitochondria and the validation of its therapeutic efficacy in a model of acetaminophen (APAP) liver injury caused by oxidative stress in mitochondria. A CoQ_10_-MITO-Porter, a mitochondrial targeting lipid nanoparticle (LNP) containing encapsulated CoQ_10_, was prepared using a microfluidic device. It was essential to include polyethylene glycol (PEG) in the lipid composition of this LNP to ensure stability of the CoQ_10_, since it is relatively insoluble in water. Based on transmission electron microscope (TEM) observations and small angle X-ray scattering (SAXS) measurements, the CoQ_10_-MITO-Porter was estimated to be a 50 nm spherical particle without a regular layer structure. The use of the CoQ_10_-MITO-Porter improved liver function and reduced tissue injury, suggesting that it exerted a therapeutic effect on APAP liver injury.

## Introduction

Mitochondria function as hubs for the integration and control of metabolic and immune systems by communicating with other organelles to maintain their individual functions and provide energy and signals^1,2^. This organelle produces reactive oxygen species (ROS) in the electron transport chain that produces adenosine triphosphate (ATP). ROS production is regulated by oxidoreductases and antioxidant pathways, and moderate levels of ROS that play a role in signal transmission, cell survival, apoptosis, differentiation and the activation of the immune system^3,4^. However, when mitochondria are unable to maintain homeostasis due to external stimulation, they generate excessive levels of ROS, thus inducing oxidative damage. Increased oxidative stress leads to mitochondrial dysfunction, resulting in premature ageing and the development of various diseases^5–7^. On this point, the delivery of antioxidant molecules to mitochondria would be a useful type of therapeutic strategy.

Delivering a drug or other molecule to mitochondria needs to reach the target organ, be taken up by cells and then transferred to an organelle^8,9^. The use of lipid nanoparticles (LNPs) for lipid-based drug delivery have the potential to overcome these challenges and practical applications like the coronavirus disease (COVID-19) vaccine are accelerating^10^. In recent years, microfluidic devices have attracted considerable interest as a technology for preparing LNPs. Formulation methods by fluidic control such as mixing lipid solutions with buffer solutions allow nanoparticles to be prepared in a single step. The use of a microfluidic device allows scale-up from laboratory to the good manufacturing practice (GMP) level and continuous mass preparation. Furthermore, determining the preparation parameters such as the total flow and flow rate ratio, which affect the degree of mixing solutions, and the selection of the solvents used make controlling the size of nanoparticles highly reproducibile^11,12^.

Coenzyme Q_10_ (CoQ_10_) is a well-known antioxidant molecule and also acts as an essential coenzyme for ATP production in mitochondria. CoQ_10_ is a poorly water-soluble molecule because of its long chain that consists of 50 carbon atoms^13^. In the field of nanoparticles, loading poorly water-soluble molecules using microfluidic devices into nanocarriers such as a poly-lactic-co-glycolic acid (PLGA) copolymer and solid dispersions are often used^14,15^. In contrast, little information is currently available on the use of phospholipids as a major component of LNPs. Encapsulating CoQ_10_ as a model drug into an LNP by a microfluidic device was reported to result in an emulsion-based solid dispersion^16^, but its application to LNPs has not been extensively investigated.

**Figure 1:**
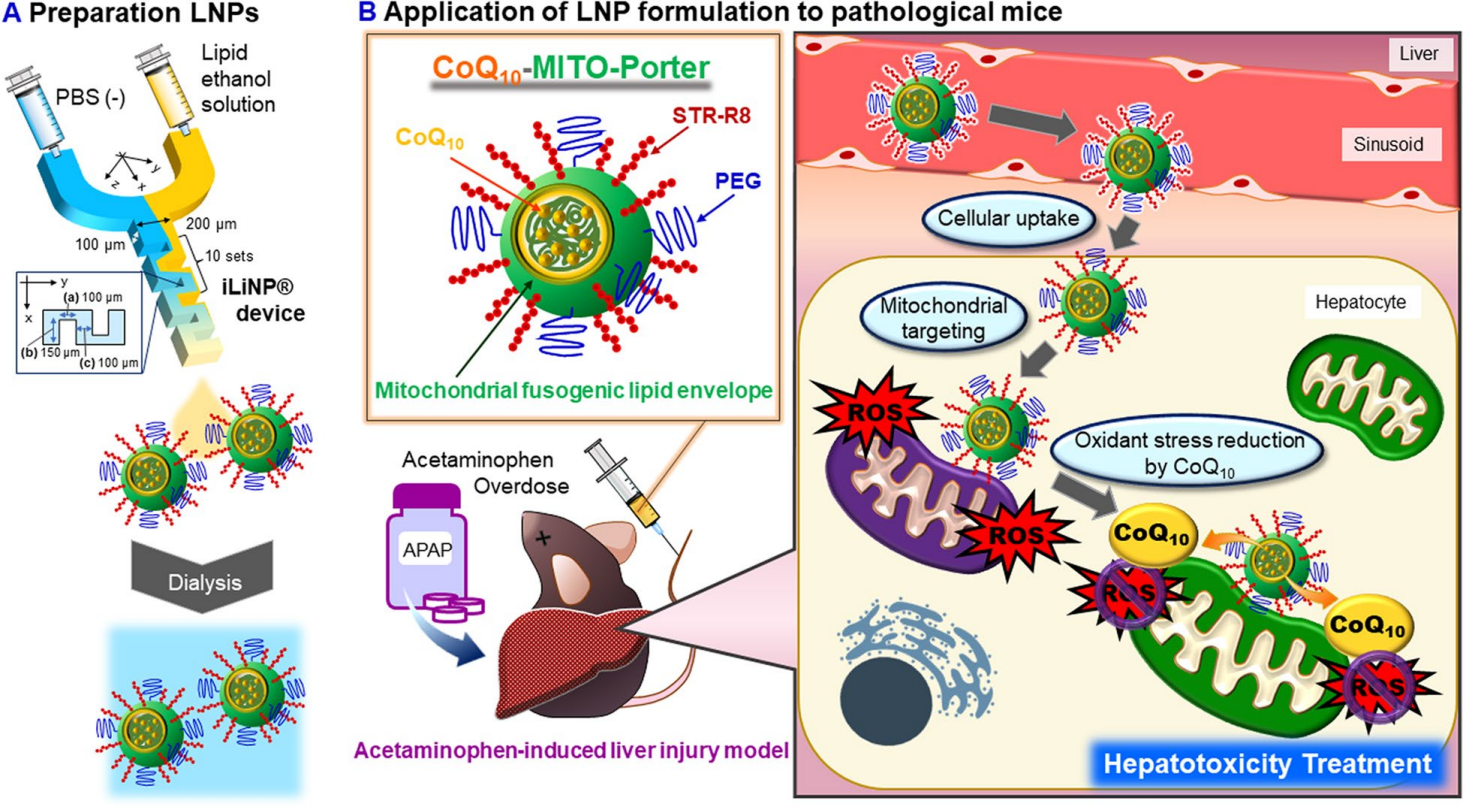
Schematic image of concept (**A**) of preparing CoQ_10_-MITO-Porter and (**B**) therapeutic strategy against APAP liver injury model using CoQ_10_-MITO-Porter. As shown in the panel (**A**), the iLiNP device contains the basic structure with 10 repetitions of baffle mixer structure. Standard dimensions of the baffle mixer structure were width (a) of 150 µm, depth (b) of 100 µm and interval (c) of 100 µm. *APAP* acetaminophen, *CoQ*_*10*_ coenzyme Q_10_, *PEG* polyethylene glycol, *ROS* reactive oxygen species, *R8* octaarginine.

We previously reported on a method for preparing a CoQ_10_-MITO-Porter, a mitochondria-targeted LNP encapsulating CoQ_10_, using a microfluidic device. The procedure had a high degree of reproducibility and could be scaled up^17^. However, the lipid composition and internal structure of the CoQ_10_-MITO-Porter was not discussed and it is important to confirm whether or not this formulation can function in vivo. In general, the internal structure of LNPs is characterized by the structure and polarity of the lipid molecules, the lipid composition ratio, and ratio of encapsulated molecules to lipids. The internal structure of the LNP may be affected by the encapsulated molecules. The present study reports on attempts to confirm that polyethylene glycol (PEG), octaarginine (R8), a functional ligand, and CoQ_10_ can be incorporated into an LNP preparation using a microfluidic device and to structurally characterize the resulting CoQ_10_-MITO-Porter by Transmission electron microscope (TEM) observations and small angle X-ray scattering (SAXS) measurements. To achieve this, a mouse model of an acetaminophen (APAP) liver injury was created to evaluate the therapeutic effect of the CoQ_10_-MITO-Porter (Fig. 1). APAP liver injury is a type of mitochondrial-related disease caused by increased oxidative stress^18,19^. The delivery of antioxidant molecules to mitochondria, the major source of ROS, has the potential to be an effective therapeutic strategy for the treatment of this type of disease. The CoQ_10_-MITO-Porter would be expected to normalize ROS levels and mitochondrial function against APAP liver injury. The biodistribution of the CoQ_10_-MITO-Porter was followed, and its therapeutic effect was evaluated using a blood biochemical test and histopathological observations.

## Results

### Effect of PEG, R8 and CoQ_10_ on the physical properties in lipid nanoparticles preparation

**Figure 2:**
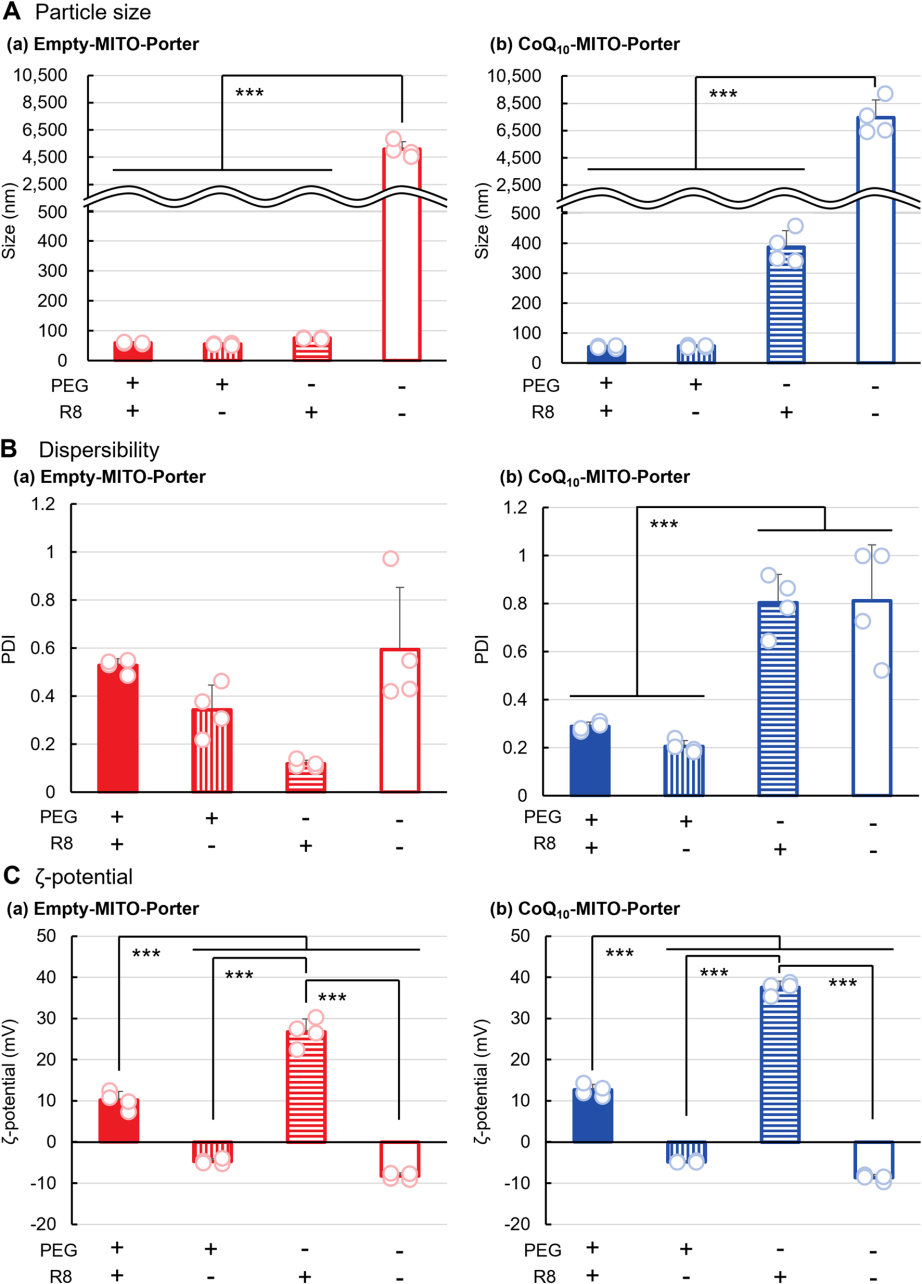
Evaluation of physical properties of the MITO-Porter with or without PEG, R8 and CoQ_10_. The prepared LNPs were characterized by three indices: (**A**) particle size, (**B**) dispersibility, (**C**) ζ-potential. The results of the physical properties of (a) Empty-MITO-Porter and (b) CoQ_10_-MITO-Porter are compared to consider the effect of CoQ_10_, a poorly water-soluble molecule. Circles represent the values of 4 individual samples and bars are the mean (n = 4). Data represented the mean ± S.D. (n = 4). Significant differences were calculated by two-way ANOVA, followed by Tukey test (***p < 0.001). In supplementary information, Table S1 shows the physical properties of the MITO-Porters.

**Figure 3:**
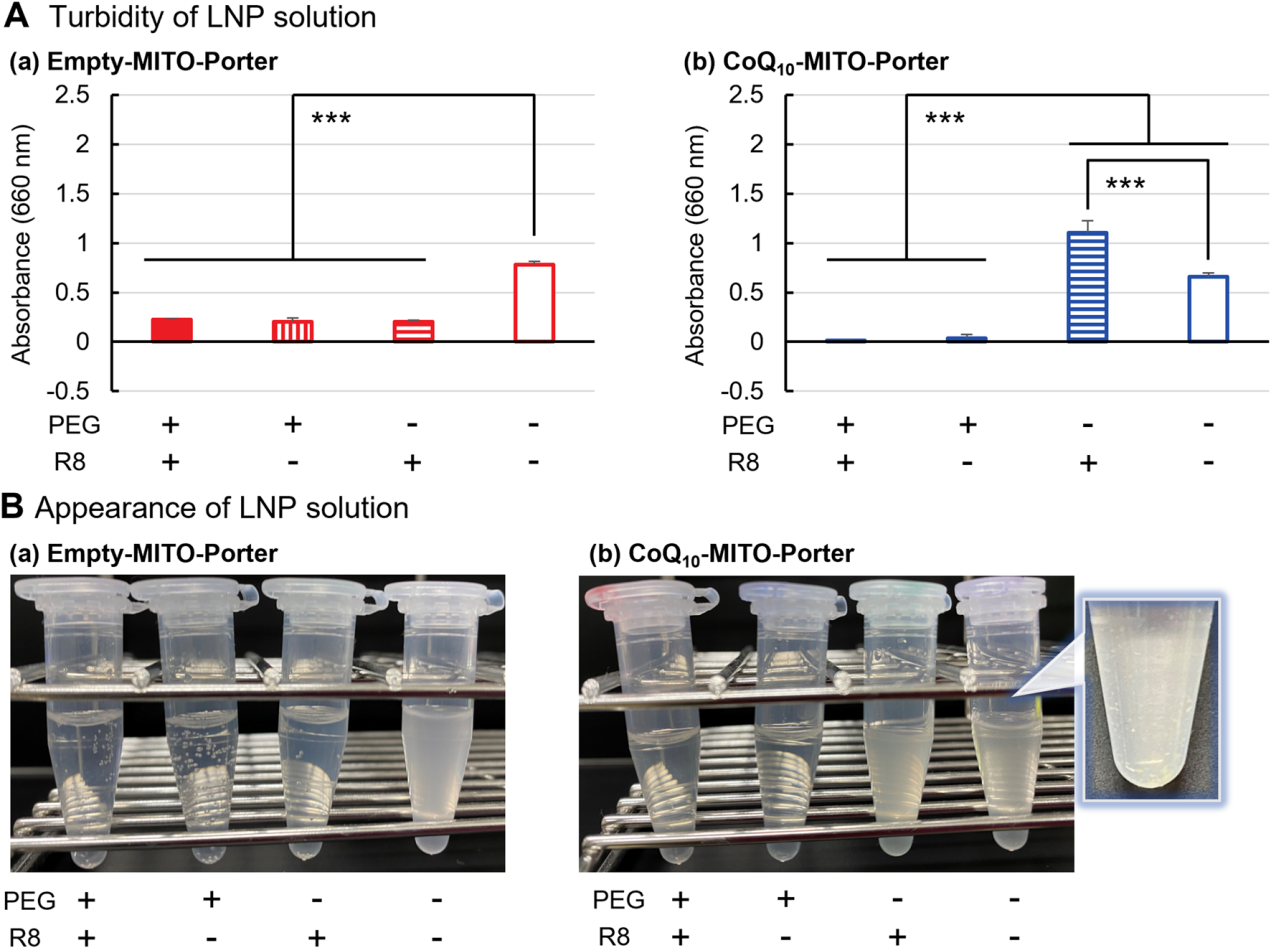
Evaluation of the appearance of the MITO-Porter solution with or without PEG, R8 and CoQ_10_. (**A**) Turbidity of LNP solution. Data represent the mean ± S.D. (n = 4). The significant differences are calculated by two-way ANOVA, followed by Tukey test (***p < 0.001). (**B**) Appearance of LNP solution. (a) Empty-MITO-Porter and (b) CoQ_10_-MITO-Porter are compared. Table S2 shows the physical properties of the MITO-Porters.

The effects of modifying PEG and R8 on the particle surface of the LNP on its properties were evaluated for 1,2-dioleoyl-*sn*-glycero-3-phosphoethanolamine (DOPE)/ sphingomyelin (SM), a MITO-Porter constituent lipid. (Figs. 2, 3). To validate the effect of the molecule that was loaded, an empty-MITO-Porter that contained no CoQ_10_, was prepared under the same conditions as were used to prepare the CoQ_10_-MITO-Porter. The MITO-Porter [PEG (−), R8 (−)] had a diameter of 1000 nm or higher and a polydispersity index (PDI) of 0.5 or higher (Figs. 2A,B, Table S1), and was suspended in a solution (Fig. 3). The CoQ_10_-MITO-Porter [PEG (−), R8 (−)] partially precipitated in the bottom of the tube (enlarged image in Fig. 3B(b)). Because of this, we concluded that these preparation conditions using [PEG (−), R8(−)] were inappropriate since they resulted in the formation of aggregates. We were able to prepare the empty-MITO-Porter by including PEG or R8 in the composition, which resulted in a clear solution of LNPs that showed interactions between the PEG and R8 (p < 0.001) (Tables 1, 2). The size of the empty-MITO-Porter [PEG (+), R8 (+)] was 59.3 ± 2.9 nm with a comparatively high PDI of 0.528 ± 0.028 (Fig. 2A(a),B(a), Table S1).

**Table 1:** Two-way ANOVA analysis of the physicochemical property of the MITO-Porters.

Sample	Parameter	Factors	P-value by 2-way ANOVA	Significant difference
Empty-MITO-Porter	Particle size	PEG	4.6 × 10^–10^	< 0.001
		R8	5.1 × 10^–10^	< 0.001
		Interaction between two factors	5.0 × 10^–10^	< 0.001
	Dispersibility	PEG	2.8 × 10^–1^	NS
		R8	6.3 × 10^–2^	NS
		Interaction between two factors	5.3 × 10^–4^	< 0.001
	ζ-potential	PEG	2.9 × 10^–5^	< 0.001
		R8	1.1 × 10^–11^	< 0.001
		Interaction between two factors	3.3 × 10^–7^	< 0.001
CoQ_10_-MITO-Porter	Particle size	PEG	5.5 × 10^–8^	< 0.001
		R8	1.5 × 10^–7^	< 0.001
		Interaction between two factors	1.5 × 10^–7^	< 0.001
	Dispersibility	PEG	1.9 × 10^–6^	< 0.001
		R8	5.8 × 10^–1^	NS
		Interaction between two factors	4.9 × 10^–1^	NS
	ζ-potential	PEG	2.0 × 10^–10^	< 0.001
		R8	4.2 × 10^–16^	< 0.001
		Interaction between two factors	5.3 × 10^–12^	< 0.001

Physicochemical property values of the MITO-Porters are performed a 2-way ANOVA analysis to compare the effect of 2 factors that are “PEG” and “R8”. A significant interaction between the two factors was found and then a simple main effect test performed. NS not significant, *p* < 0.001 by simple main effect test, followed by Tukey test.

**Table 2:** Two-way ANOVA analysis results of turbidity of the MITO-Porter solution.

Sample	Factors	P-value by 2-way ANOVA	Significant difference
Empty-MITO-Porter	PEG	4.7 × 10^–13^	< 0.001
	R8	3.6 × 10^–11^	< 0.001
	Interaction between two factors	7.3 × 10^–13^	< 0.001
CoQ_10_-MITO-Porter	PEG	4.8 × 10^–8^	< 0.001
	R8	8.3 × 10^–3^	< 0.01
	Interaction between two factors	8.9 × 10^–3^	< 0.01

Absorbance intensity of the MITO-Porters are performed a 2-way ANOVA analysis to compare the effect of 2 factors that are “PEG” and “R8”. A significant interaction between the two factors was found and then a simple main effect test performed. *p* < 0.001 by simple main effect test, followed by Tukey test.

The combination of PEG and R8 conferred positive effects on the properties of the CoQ_10_-MITO-Porter (p < 0.001) (Tables 1, 2). The size of the CoQ_10_-MITO-Porter [PEG (−), R8 (+)] tended to decrease: 387.9 ± 54.2 nm compared to CoQ_10_-MITO-Porter [PEG (−), R8 (−)], but the PDI value was high: 0.804 ± 0.119 (Fig. 2A(b),B(b), Table S1), becoming an opaque solution (Fig. 3A(b),B(b)). R8 alone was not sufficient to stabilize the dispersion of the CoQ_10_-MITO-Porter. The use of PEG (resulting in the formation of small-sized particles, and in stabilizing the dispersion) were essential for successfully preparing the CoQ_10_-MITO-Porter. The resulting CoQ_10_-MITO-Porter [PEG (+), R8 (+)] had the following values: particle size: 54.4 ± 4.8 nm and PDI: 0.289 ± 0.0188, which were acceptable features for such an LNP. The ζ-potential of the LNPs showed similar behavior regardless of the presence of CoQ_10_. [PEG (+), R8 (−)] and [PEG (−), R8 (+)] are negatively and positively charged, respectively. [PEG (+), R8 (+)] was charged at approximately 10 mV (Fig. 2C).

Preparing the LNP using a microfluidic device required the inclusion of PEG or R8 in the lipid composition to ensure particle dispersion. We therefore concluded that PEG is essential for preparing LNPs that contain poorly water-soluble molecules such as CoQ_10_.

### Evaluation of the internal structure of CoQ_10_-MITO-Porter particles

**Figure 4:**
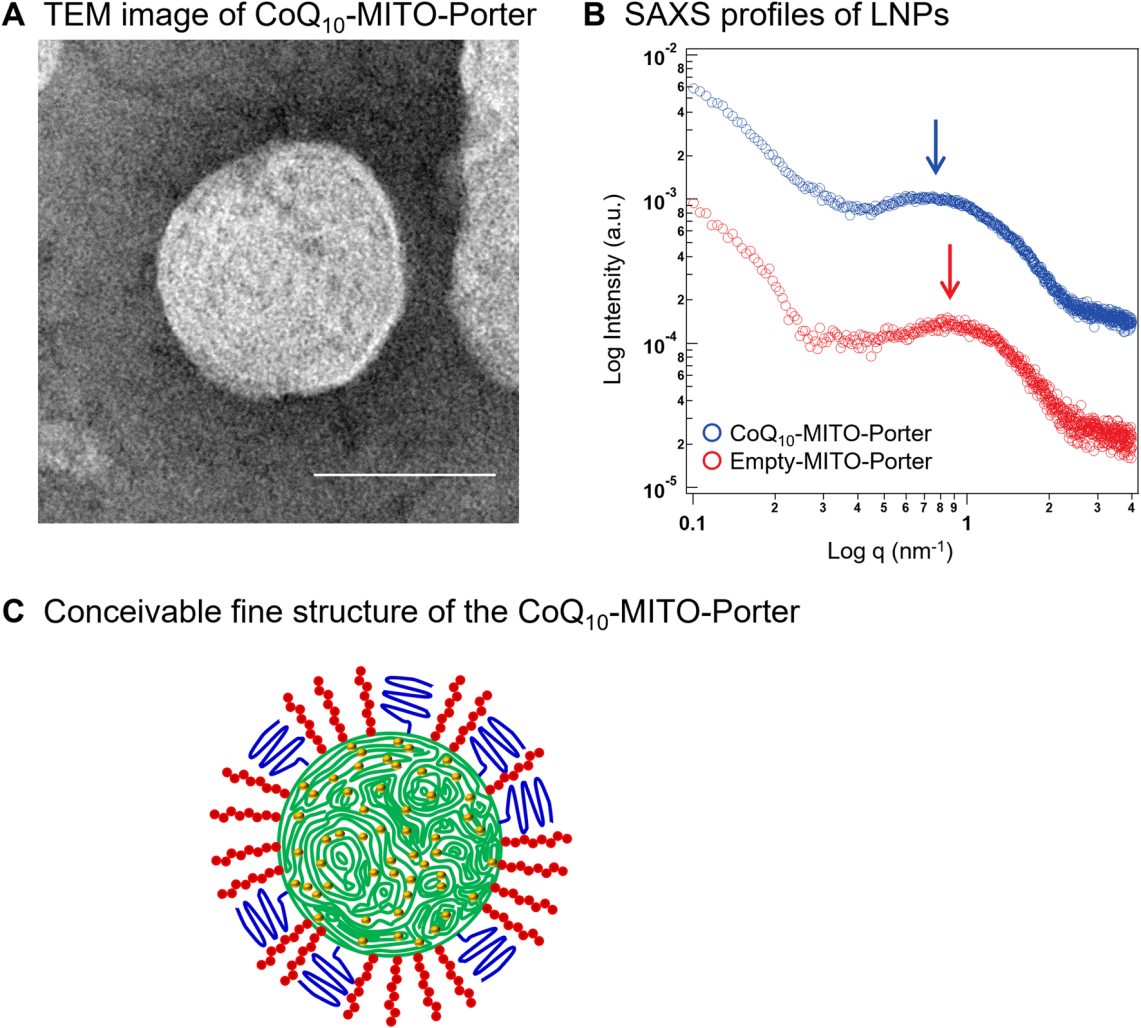
Evaluation of the internal structure of CoQ_10_-MITO-Porter particles. (**A**) TEM image of CoQ_10_-MITO-Porter. Scale bar 50 nm. (**B**) SAXS profiles of LNPs. Red and blue dot plots show (**A**,**B**), respectively. (**C**) Conceivable fine structure of the CoQ_10_-MITO-Porter. Table S5 shows the physical properties of the MITO-Porters used for SAXS measurements.

The structure of the CoQ_10_-MITO-Porter was evaluated after being prepared by a microfluidic device and after dialysis. TEM observations showed that the CoQ_10_-MITO-Porter is a spherical particle with a diameter of approximately 50 nm (Fig. 4A). SAXS measurements were employed to characterize the fine internal structure of the particles (Fig. 4B). In general, the scattering intensity profiles obtained from this measurement method are useful for predicting the size distribution, shape and structure of particle samples. The CoQ_10_-MITO-Porter and the empty-MITO-Porter were used to compare whether encapsulating CoQ_10_ or not makes a difference to the morphology of the LNP. Broad peaks appeared at 0.8 nm^−1^ for CoQ_10_-MITO-Porter (blue profile in Fig. 4B) and at 0.9 nm^−1^ for the empty-MITO-Porter (red profile in Fig. 4B). In SAXS measurements, nanoparticles with a lamellar structure and nanoparticles without a periodic structure show a sharp peak and a broad peak, respectively^20,21^. The CoQ_10_-MITO-Porter and the empty-MITO-Porter were inferred to be tight structures with no lamellar phase based on the appearance of broad peaks. These results were consistent with the TEM observations. The drug/lipid (w/w) of CoQ_10_-MITO-Porter is 0.19 ± 0.03^17^. The fine structure of the MITO-Porter was not changed by the presence of CoQ_10_. The position of the peak reflects the particle size. The smaller the particle, the more the peak shifts towards the wide-angle side^22^. The peak for the empty-MITO-Porter was shifted towards the wide-angle side compared to the CoQ_10_-MITO-Porter, suggesting a smaller diameter (Table S5). This is also consistent with our evaluation of the physical properties of the LNPs.

Based on the results of TEM observations and SAXS measurements, a conceivable image of fine structure of the CoQ_10_-MITO-Porter was shown in Fig. 4C. These results suggest that the CoQ_10_-MITO-Porter is a spherical particle with a non-lamellar interior form of approximately 50 nm. The fine structure of the LNPs would be expected to ensure that the molecule functions as a drug, because of the stability and the release of the formulation.

### Tissue migration of CoQ_10_-MITO-Porter

**Figure 5:**
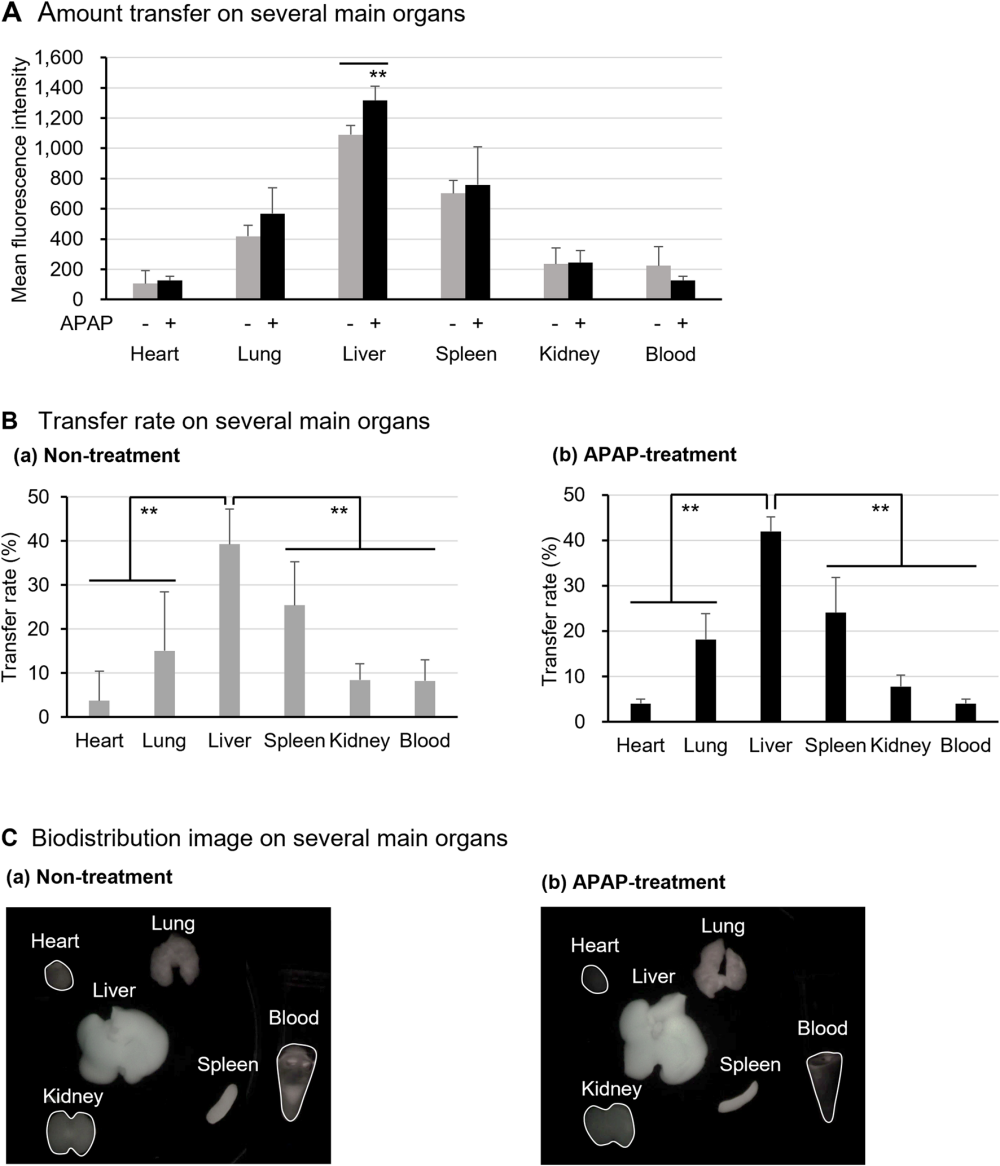
Biodistribution of the CoQ_10_-MITO-Porter in mice. Ex vivo images of organs are obtained from non-treated or APAP-treated mice 3 h after injection with DiD-labeled CoQ_10_-MITO-Porter. (**A**) Amount transferred to several main organs. Based on the observed images, fluorescence intensity is quantified using image J and defined as the amount of transferred to the organs. Data represent the mean ± S.D. (n = 3). Significant differences were calculated by the Unpaired t-test (**p < 0.01). (**B**) Transfer rate on several main organs. The transfer rate is calculated from the transfer amount results. Data represent the mean ± S.D. (n = 3). The significant differences (vs Liver) were calculated by one-way ANOVA, followed by SNK test (**p < 0.01). (**C**) Biodistribution image on several main organs. (a) Non-treatment. (b) APAP-treatment. Table S6 shows the physical properties of the administered CoQ_10_-MITO-Porter.

The biodistribution of the CoQ_10_-MITO-Porter was evaluated by ex vivo imaging systems in normal and APAP liver injury model mice (Fig. 5). The amount transferred and rate of transfer into each tissue were calculated based on the observed images. Several major tissues were observed at 3 h after the intravenous administration of the 1,1'-dioctadecyl-3,3,3',3'- tetramethylindodicarbocyanine, 4-chlorobenzenesulfonate salt (DiD)-labelled CoQ_10_-MITO-Porter (Fig. 5C). In the case of the APAP liver injury model mice, the CoQ_10_-MITO-Porter was administrated at 1 h after the APAP treatment. The CoQ_10_-MITO-Porter accumulated at the highest levels in the liver among the major tissues. The accumulation of the LNPs in the APAP-induced liver injury group was significantly increased compared to the non-treated group (Fig. 5A). The CoQ_10_-MITO-Porter was delivered to the liver, followed by the spleen, lung, kidney, and heart. The APAP-treated mice tended to lose the CoQ_10_-MITO-Porter from the blood. No differences between the two groups were found in the transfer rate into each tissue. The transfer rate of the LNPs to the liver was approximately 40%.

Accordingly, the CoQ_10_-MITO-Porter appears to be transferred to the liver by intravenous administration, meaning that it could be applied to the treatment of liver diseases such as an APAP liver injury.

### Evaluation of the therapeutic effect of the CoQ_10_-MITO-Porter against the APAP liver injury model

**Figure 6:**
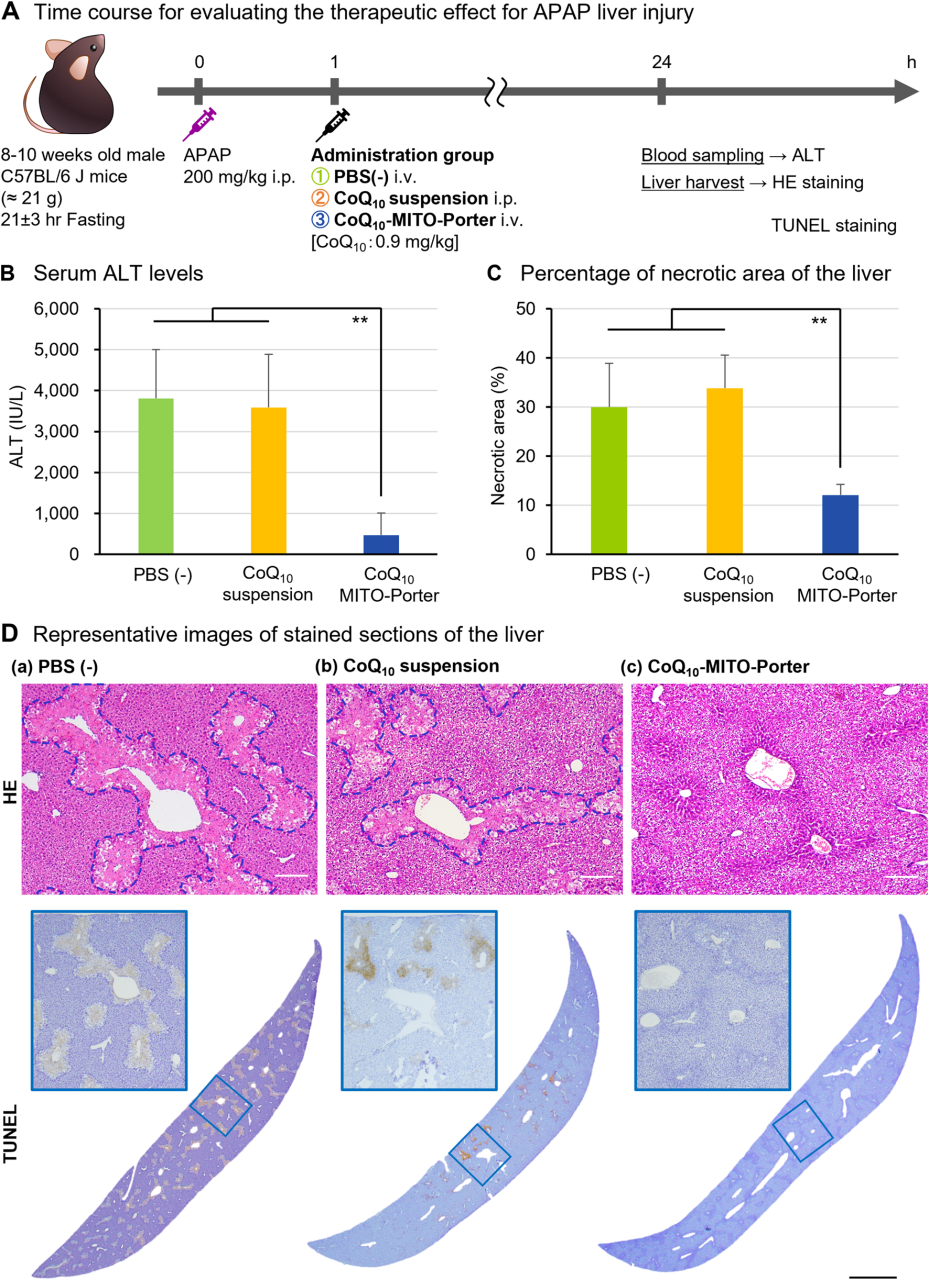
Confirmation that the delivery of CoQ_10_ using the MITO-Porter system protects against APAP-induced liver injury. Schematic diagrams show (**A**) Time course for evaluating the therapeutic effect for APAP liver injury animal studies. Mice were injected with 200 mg/kg APAP intraperitoneally and after 1 h, with CoQ_10_-MITO-Porter and PBS (−) intravenously or CoQ_10_ suspension intraperitoneally. The CoQ_10_ dose was kept evenly at 0.9 mg/kg. The blood and liver from each mouse were collected at 24 h later from APAP-treatment. (**B**) Serum ALT levels. Data represent the mean ± S.D. (n = 3–6). Figure S3 showed serum LDH. Figure S4 describes a control experiment in which the empty-MITO-Porter was used. (**C**) Percentage of necrotic area based on the HE-stained whole liver sections. Data are displayed as the mean ± S.D. (n = 6–10). Panel (**D**) show representative images of stained sections of the liver: (a) PBS (−), (b) CoQ_10_ suspension, (c) CoQ_10_-MITO-Porter group. In HE-stained sections, dashed line encloses necrotic area. Scale bars are 100 µm. In TUNEL-stained sections, scale bar is 1000 µm. The significant differences (vs CoQ_10_-MITO-Porter group) were calculated by one-way ANOVA, followed by SNK test (**p < 0.01). The physical properties of the administered CoQ_10_-MITO-Porter are reported Table S7 in supplementary information.

The therapeutic effect of the CoQ_10_-MITO-Porter was tested against an APAP-induced liver injury (Fig. 6). APAP is oxidized by CYP2E1 to the active metabolite *N*-acetyl-*p*-benzoquinone imine (NAPQI) in the liver. NAPQI increases the oxidative stress associated with mitochondrial dysfunction, developing liver damage. Since CYP2E1 is a cytochrome P450 molecule, a drug-metabolizing enzyme in every animal species, APAP liver injury can be reproduced by a single dose in experimental animals, which cause acute toxicity symptoms that are very similar to that in humans. In the case, the drug-induced liver injury model was created as a system for evaluating drug-induced liver injury, and was used to clarify the mechanisms of hepatotoxicity and to evaluate the therapeutic effect of the preparation. Mice with APAP hepatotoxicity can be produced with mild symptoms: serum alanine aminotransferase (ALT) levels < 1000 IU/L and moderate models: serum ALT levels ~ 3000 IU/L according to the feeding conditions and the drug loading dose, they were often assessed at an early stage: 6–8 h, an intermediate stage: 12 h and a late stage: 24 h after the APAP treatment^23–26^. In the present experiment, 24 h after injection was selected as the evaluation time to evaluate the persistence of the protective effect of CoQ_10_-MITO-Porter against a moderate model of APAP liver injury (Fig. 6A).

C57BL/6 J mice (10 weeks old, ~ 21 g, fasted for 21 ± 3 h) were given 200 mg/kg APAP intraperitoneally. Samples: a phosphate-buffered saline (PBS) (−), CoQ_10_ suspension (0.9 mg/kg CoQ_10_) and the CoQ_10_-MITO-Porter (0.9 mg/kg CoQ_10_) were administered at 1 h post-injection and serum ALT values were measured 24 h later (Fig. 6B). The results showed higher serum ALT levels, in the PBS (−) group: 3,812.2 ± 1190.6 IU/L and the CoQ_10_ suspension: 3,590.3 ± 1298.0 IU/L. In contrast, the CoQ_10_-MITO-Porter group significantly relieved liver damage, serum ALT values 470.3 ± 538.5 IU/L. The value of serum lactate dehydrogenase (LDH) also showed a similar trend to that for serum ALT (Fig. S3). In a preliminary study, the empty-MITO-Porter was, as expected, found to have no hepatoprotective effect (Fig. S4). The therapeutic effect was assessed by liver histology using hematoxylin and eosin (H&E) stains and terminal deoxynucleotidyl transferase dUTP nick end labeling (TUNEL) stains, the degree of tissue damage and the presence of apoptosis, respectively (Fig. 6D). In HE-stained sections of the PBS (−) group and the CoQ_10_ suspension group, a necrotic area extended around the portal vein (blue dotted line in Fig. 6D) and morphological abnormalities such as loss or shrinkage of the nucleus and cell swelling were observed. On the contrary, few necrotic images were observed in the CoQ_10_-MITO-Portert group and hepatocyte sequence regularity was maintained. Percentage of necrotic area was quantitatively determined based on HE-stained images (Fig. 6C). The quantitative results showed significant differences between the CoQ_10_-MITO-Porter group and the other groups. In TUNEL-stained sections of the PBS (−) and CoQ_10_ suspension groups, brown apoptosis-positive regions were observed in extensive areas of the liver, indicating that cell death was induced by oxidative stress caused by APAP hepatotoxicity. A few brown areas were observed in the CoQ_10_-MITO-Portert group. Thus, the histological evaluation also showed that CoQ_10_-MITO-Porter reduced APAP liver injury.

The results from these animal experiments indicate that administration of CoQ_10_-MITO-Porter is effective in APAP liver injury by delivering the antioxidant CoQ_10_ to the liver, thereby reducing oxidative stress and restoring tissue damage and biochemical functions.

## Discussion

APAP liver injury is triggered by mitochondria-induced oxidative stress. The application of antioxidant molecules to APAP-damaged liver injury is considered to be an effective preventive and therapeutic strategy. Previous studies have attempted to use CoQ_10_, resveratrol and 2-(2,2,6,6-tetramethylpiperidin-1-oxyl-4-ylamino)-2-oxoethyl) triphenylphosphonium chloride (Mito-TEMPO). For example, in an experimental system in which APAP liver injury model mice (C57BL/6 J, male, 8 weeks old, fasting) were administered CoQ_10_ solution (5 mg/kg CoQ_10_ in a soybean lecithin solution, intraperitoneal administration) at 1.5 h after APAP administration, the serum ALT levels in the PBS (−) group were approximately 2400 IU/L, whereas that in the CoQ_10_ group they were approximately 1500 IU/L, suggesting improved liver function^24^. The CoQ_10_-MITO-Porter (0.9 mg/kg CoQ_10_, intravenously administered at 1 h post-APAP injection) showed significantly decreased serum ALT levels, indicating that it suppressed liver injury (Fig. 4B). The CoQ_10_-MITO-Porter produced a therapeutic effect at CoQ_10_ concentrations as low as 0.9 mg/kg. We therefore conclude that, based on the therapeutic evaluation, that the particle designs of the LNPs, which contained soluble forms of CoQ_10_ and the regulation of the CoQ_10_ pharmacokinetics, allowed the particles to be transferred to liver mitochondria following its introduction into hepatocytes in the liver. The use of microfluidic devices as a method for preparation also largely contributed to the formation of stable LNPs as small as 50 nm, which was difficult using the batch method. The purpose of the present study was to examine the therapeutic effects of CoQ_10_ delivery to mitochondria to alleviate oxidative stress levels by eliminating mitochondrial dysfunction. The major differences between the findings reported in the previous study and the current study are the size of the LNPs and the dose of CoQ_10_. In general, it is more difficult to prove that a treatment is effective than to prove a prevention effect. A previous study demonstrated the hepatoprotective effect of the preventive administration of a conventional 80 nm CoQ_10_-MITO-Porter (2 mg/kg CoQ_10_) in a mouse model of hepatic ischemia-reperfusion^27^. The formulary difference in the novel CoQ_10_-MITO-Porter was that low CoQ_10_ dosages could provide significant therapeutic effects with low burdens to the body.

In the first step in the preparation of LNPs an invasive lipid nanoparticle production (iLiNP®) device, which contains a flow channel structure (10 sets of baffle mixer structures) and flow conditions (Total flow = 500 µL/min, Flow rate ratio = 4) in the microfluidic device, enable the rapid mixing of lipids/ethanol solutions and buffer solutions, thus leading to the formation of small-sized nanoparticles^28^. Specifically, this led to a shorter growth process by fusion and aggregation of the lipid-associated complex, namely, the lipid core, the lipid disc and LNP formation. Secondly, during the dialysis process, in solutions containing 20% ethanol, the LNPs tended to restructure, maintaining a thermodynamically stable dispersion in aqueous solution according to their lipid properties and composition. The MITO-Porter [PEG (−), R8 (−)], composed of neutral lipids DOPE/SM, self-assembles through intermolecular hydrophobic interactions. However, the thermodynamically unstable particles formed aggregates under the present preparation conditions using a microfluidic device, that produces a rapid polarity change with dilution of the ethanol solution, and the dialysis process completely replaces the aqueous solvent. As a result, CoQ_10_ was precipitated out when it reached a supersaturated state (Figs. 1, 2, S1, S2).

The charged PEG and R8 are oriented on the LNP surface, creating an electrostatic repulsive force that inhibits fusion and aggregation between particles. The MITO-Porter [PEG (+), R8 (+)] possessed a zeta potential of approximately 10 mV that was derived from R8 (10 mol% of total lipids), that causes the LNPs to be stabilized by electrostatic repulsive forces. PEG is located on the outer layer of the LNP, exerting a protective colloidal action to form a hydration layer and a size-controlling effect^29^. In the CoQ_10_-MITO-Porter [PEG (+), R8 (+)] caused the formation of stable particles due to the effect of PEG. On the other hand, the empty-MITO-Porter [PEG (+), R8 (+)] could also be prepared, but its PDI was higher than that of the CoQ_10_-MITO-Porter. After a microfluidic device preparation, the particle size was very small, 34.2 ± 6.7 nm (PDI = 0.257 ± 0.0187) (Fig. S1, Table S1). Thermodynamically unstable nanoparticles tended to aggregate because, in small-sized nanoparticles with a high curvature there was a substantial distance between the lipid molecules, allowing easy contact between hydrophobic regions of lipids and water molecules during the dialysis. The PEG and R8 ratios in the present study are the optimized conditions for the preparation of CoQ_10_-MITO-Porter. An Empty-MITO-Porter should be optimized to ensure dispersion stability.

Preparing LNPs requires the inclusion of materials in the final product that inhibit the excessive self-assembly of lipids and provide for the formation of a stable dispersion system. PEG was found to be a particularly important factor in enhancing dispersion stability and to efficiently package for poorly water-solubility molecules such as CoQ_10_. PEG can also control pharmacokinetics including blood retention and physicochemical properties of the formulation by differences in the head structure and lipid scaffold structure^30^. To achieve purposeful LNP preparation an adequate PEG should be used and the ratios should be optimized.

Biodistribution evaluations showed that CoQ_10_-MITO-Porter largely accumulated in the liver after intravenous administration. In the case of induced liver injury, the delivery of LNPs to the liver was increased significantly (Fig. 5). CoQ_10_-MITO-Porter without R8 modification significantly decreased accumulation in liver and liver mitochondria^27^. The CoQ_10_-MITO-Porter was modified with R8 on the particle surface. The data for the resulting CoQ_10_-MITO-Porter were in agreement with previous reports in that R8-modified nanoparticles tend to accumulate in the liver^31^. The delivery of therapeutic molecules to hepatocytes is an important therapeutic strategy for the treatment of an APAP liver injury in order to reduce the levels of NAPQI, a toxic metabolite of APAP, in hepatocytes^18,19^. The capillary walls of the liver contain numerous small pores called fenestra. Therefore, the LNPs must pass through the fenestra and eventually reach hepatocytes. Since the fenestra diameter averages 141 nm and 107 nm for mice and humans, respectively^32^, the particle size needs to be controlled to less than 100 nm. The administration of 60 nm nanoparticles to mice caused a reduced APAP hepatotoxicity by allowing them to pass through the fenestra and easily access hepatocytes, as has been previously reported^33^. From our previous studies, an 84 nm CoQ_10_-MITO-Porter has been observed in hepatocytes when administered intravenously to mice^27^. The 50 nm CoQ_10_-MITO-Porter used in this study was also expected to be transferred to hepatocytes similar to the previous study. In APAP liver injury, sinusoidal endothelial cells are damaged before hepatocytes, leading to the disruption of the sinusoidal wall and the mobilization of leukocytes to the inflammation sites^34^. The nanoparticles that were modified with R8 were opsonized in the liver and recognized by macrophages^29^. In Fig. 5, mice with an APAP liver injury model likely have increased LNP migration compared to normal mice because of the enlargement of fenestra and the increased mobilization of hepatic macrophages. Moreover, it will be necessary to check whether CoQ_10_ is delivered to mitochondria by tracking the particle dynamics in the future. Lipid-based drug delivery contributes to the achievement of therapeutic strategies by the selection of ligands with an affinity for specific target tissues, cells, and cell organelles and the control of particle size.

The use of the CoQ_10_-MITO-Porter in the APAP liver injury model resulted in reducing oxidative stress, namely, improved ALT levels, decreased areas of tissue necrosis, and suppressed apoptosis (Fig. 6). Serum ALT levels were correlated with the histologic evaluation. The CoQ_10_-MITO-Porter group exhibited significantly lower percentage of necrotic area than the PBS (−) group and the CoQ_10_ suspension group (p < 0.01). Although NAPQI is produced at optimal doses, the detoxification mechanism functions to inactivate it by using GSH. An APAP overdose in mice causes the depletion of GSH within 30 min, after which, the NAPQI combines with intracellular proteins, and the maximum amount of NAPQI-protein complex is reached within 30 min to 1 h^23^. NAPQI binds to mitochondrial proteins and induces mitochondrial dysfunction and destruction due to increased oxidative stress, which then results in the release of apoptosis-inducing factors (AIF), such as endonuclease G and cytochrome c from mitochondria, followed by the initiation of DNA damage and necrotic cell death^18,19^. These phenomena were clearly observed in both the PBS (−) group and CoQ_10_ suspension group. For this reason, the administration of CoQ_10_-MITO-Porter 1 h after APAP loading constitutes therapeutic treatment.

Regarding therapeutic targeting organelles for APAP liver injury, mitochondria are attractive because they play central roles in the signaling of numerous important cellular events^19^. ROS are primarily produced by the electron transfer system that is located in the mitochondrial inner membrane^18^. It would therefore by expected that delivering antioxidant molecules such as CoQ_10_ to the interior of mitochondria would effectively scavenge excessive ROS caused by oxidative stress. It was previously shown that a radio isotope-labeled CoQ_10_-MITO-Porter accumulates in liver mitochondria^27^. In a previous study, hepatoprotective effects were compared between LNPs with different mitochondrial fusion activity in a mouse model of hepatic ischemia-reperfusion^27^. The CoQ_10_-MITO-Porter improved ALT levels, but the CoQ_10_-loaded LNP (CoQ_10_-EPC-LP) used in that study was composed of mitochondrial low fusion lipids, showed higher levels. In the evaluation of mitochondrial fusion activity using these two LNPs, the CoQ_10_-MITO-Porter showed fusion activity, while CoQ_10_-EPC-LP had little fusion ability. It is possible that the CoQ_10_-MITO-Porter in the present study has mitochondrial fusion properties because it has the same lipid composition as the LNPs used in the previous study. CoQ_10_ functions as a coenzyme for ATP production in the mitochondrial electron transfer system, and would be expected to improve mitochondrial function. The mechanism responsible for the therapeutic effect was not fully elucidated in this study. Additional studies will be needed to evaluate oxidative stress markers and mitochondrial function. The delivery of CoQ_10_ to mitochondria by the MITO-Porter system is one of the important therapeutic strategies against mitochondria-related diseases such as APAP liver injury that is associated with oxidative stress.

## Methods

### Materials

DOPE and 1,2-dimyristoyl-*rac*-glycero-3-methylpolyoxyethylene 2000 (DMG-PEG 2000) were purchased from the NOF Corporation (Tokyo, Japan). SM derived from porcine brain was purchased from Avanti Polar Lipids (Alabaster, AL, USA). Ubiquinone-10 (CoQ_10_) was purchased from FUJIFILM Wako Pure Chemical Co (Osaka, Japan). Stearyl-R8 was purchased from Toray Industries, Inc (Tokyo, Japan). DiD was purchased from Funakoshi Co., Ltd (Tokyo, Japan). PBS (−) tablets were purchased from Takara Bio Inc (Shiga, Japan).

### Experimental animals

C57BL/6 J male mice (8–10 weeks old, ~ 21 g) were purchased from Hokudo Co., Ltd (Sapporo, Japan). The mice were housed at a 12 h light–dark cycle with free intake of water and standard mouse food in a specifically pathogen-free room, acclimated to the breeding environment for a week and then received each treatment. From an animal welfare concept, it was decided to evaluate the small number of cases for which a statistical analysis could be performed. Euthanasia was performed by cervical dislocation while isoflurane inhalation anesthesia was used in cases of sufficient activity. All animal protocols were approved by the institutional Animal Care and Research Advisory Committee of the Faculty of Pharmaceutical Sciences, Hokkaido University, Sapporo, Japan (date: March 17, 2022, registration no. 18-0096).

### Preparation of the CoQ_10_-MITO-Porter

The CoQ_10_-MITO-Porter was prepared by a microfluidics system using a iLiNP® device (basic structure of 10 baffle mixer structure sets, standard dimensions of the baffle mixer structure: a width of 150 μm, a depth of 100 μm and an interval of 100 μm). 4.2 mM lipids (DOPE:SM:DMG-PEG2000:STR-R8 [9:2:0.33:1.1, molar ratio]) and 0.7 mM CoQ_10_ were dissolved in ethanol. For the fluorescent labeling, DiD was added to the lipid solution at 0.5 mol% per amount of total lipid. The lipid solution was diluted with PBS (−) in the mixing site of the iLiNP device at total flow of 500 µL/min [flow rare ratios (water: ethanol) = 4: 400 µL/min for PBS (−) and 100 µL/min for the lipid phase]. Syringe pumps (Harvard Apparatus, Holliston, MA) were used to control the flow rate. The resulting sample was subsequently dialyzed for at least 2 h against PBS (−) dialysis using Spectra/Por 4 dialysis membranes (molecular weight cutoff 12,000–14,000 Da; Spectrum Laboratories, Rancho Dominguez, CA). The size, PDI, ζ-potentials of the MITO-Porters were measured by Dynamic Light Scattering (DLS) using a Malvern Zetasizer Nano ZS instrument (Malvern Instruments Ltd., Malvern, UK). The PDIs indicate of particle size distribution, ranged from 0 to 1. To quantify the appearance of the LNP solution, the absorbance of the MITO-Porters solution was measured with a DU-730 (Beckman Coulter, Brea, CA, USA). The measurement wavelength was set to 660 nm, which is not affected by the color tone. In animal studies, concentrated LNP solutions were administered. In animal studies, concentrated LNP solutions were administered. A volume of 1.5 mL of the prepared suspension was added to 8.5 mL PBS (−) followed by ultrafiltration through an Amicon® system (Millipore, Billerica, MA, USA) to make LNP solution concentrated (a final volume of 200 µL per a set). Determination of lipid and CoQ_10_ concentration was performed using established protocols as previously described^17^.

### TEM observations

TEM observations of the MITO-Porters were carried out at the Tokai Electron Microscopy, Inc. (Aichi, Japan). The sample solution was dropped on a copper grid covered with a carbon film and washed with distilled water. After dropping a 2% solution of phosphotungstic acid (pH 7.0) on the grid, the samples were observed by JEM-1400Plus (JEOL Ltd., Tokyo, Japan) with negative stain.

### SAXS measurement

SAXS measurements were performed on the beamline BL15A2 at the Photon Factory (Tsukuba, Japan). LNP suspensions were introduced into a flow focusing-type of microfluidic device made from a cyclic olefin polymer to avoid X-ray radiation damage during the measurement. A wavelength was 1.2 Å, and a SAXS detector (PILATUS3 2 M, DECTRIS, Switzerland) distance was set to 1.5 m. SAXS data were collected with 1 s exposure time and integrated 1,200 images.

### Biodistribution of the CoQ_10_-MITO-Porter

The mice were fasted for 21 ± 3 h pre first injection and allowed only drinking water. The non-treated and APAP-treated groups received PBS (−): 10 µL/g and APAP solution: 200 mg/10 mL/kg intraperitoneally, respectively, and 1 h later, both groups were injected intravenously with DiD-labeled CoQ_10_-MITO-Porter at a lipid dosage of 20 nmol/8 µL/g. Three hours after LNP administration, the mice were collected, whole blood was obtained by cardiac blood sampling and sacrificed. Subsequently, the heart, lung, liver, spleen, and kidneys were harvested immediately, washed with PBS (−). The organs were imaged using a FluorVivo™ 300 (INDEC BioSystems, Los Altos, CA, USA). DiD was excited with a 644 nm light, and the red filter of the fluorescence detection channel was set for DiD. All images were analyzed using Image J software (http://rsb.info.nih.gov/ij/). The transfer rate on several main organs was calculated as follows: quantified fluorescence efficiency of an objective organ/ quantified fluorescence efficiency of all organs × 100.

### Therapeutic effect validation against APAP liver injury model mice

For determining the therapeutic effects of CoQ_10_-MITO-Porter, mice were randomly divided into 3 groups of 6, 3 and 3 mice, named PBS (−), CoQ_10_ suspension and CoQ_10_-MITO-Porter groups, respectively. APAP was dissolved in PBS (−), heated at 60 °C for 30 min. The CoQ_10_ suspension was prepared by dispersing in PBS (−), sonicated and heating at 50 °C for 5 min. Samples containing CoQ_10_ were administered to mice at a dose of 0.9 mg/kg CoQ_10_ in a total volume of 8 mL/kg. The fasted mice were injected intraperitoneally with 200 mg/kg APAP. After 1 h, The PBS (−) and CoQ_10_-MITO-Porter groups were administered intravenously and the CoQ_10_ suspension group intraperitoneally. At 6 h after APAP treatment, blood was collected from the mice, followed by euthanasia. To measure serum ALT levels, a marker of liver function, blood was incubated at room temperature for 1 h and serum was obtained by centrifugation (800*g*, 4 °C, 5 min). Serum ALT value was determined by Pure Auto S ALT-L kit (Sekisui Medical Co., Ltd, Tokyo, Japan). For the evaluation of the areas of necrosis and DNA fragmentation, the liver was harvested immediately after euthanasia, washed with PBS (−), and stored in 4% (v/v) paraformaldehyde in PBS (−) for 48 h at 4 °C. Paraffin-embedded liver section, HE staining and TUNEL staining were performed by Morphotechnology Co., Ltd (Sapporo, Japan). Stained liver sections were observed with the SMZ 1270i (Nikon Corporation, Tokyo, Japan). HE-stained sections were analyzed to identify percentage of necrotic areas using Image-pro Plus 7.0. The percentage of necrosis was calculated as the ratio of necrotic areas to whole tissue areas.

### Statistical analysis

The data shown in Figs. 2, 3A, 5A,B, 6B,C are expressed as the mean ± standard deviation (S.D.) for the indicated number of experiments. In Figs. 2 and 3A, we performed 2-way ANOVA, followed by Tukey test to compare the effect of 2 factors. If a significant interaction between the two factors was found, a simple main effect test was also performed. In Fig. 5A, the statistical significances between 2 groups were examined by the unpaired t-test. In Figs. 5B, 6B,C for multiple comparisons, we performed 1-way ANOVA, followed by the SNK test. A difference with p < 0.05 was considered to be statistically significant.

### Animal experiments

The use of the mice was approved by the Ethics of Pharmaceutical Science Animal Committee of Hokkaido University (approval number: 18-0096). All experiments were performed in accordance with National University Corporation Hokkaido University Regulations on Animal Experimentation and with the ARRIVE guidelines.

## Supplementary Information

Supplementary Information.

## Data Availability

The datasets used and/or analyzed during the current study available from the corresponding author on reasonable request.
