# Supplementary material for: A system that delivers an antioxidant to mitochondria for the treatment of drug-induced liver injury
Source: Sci Rep. 2023 May 10;13:6961. doi: 10.1038/s41598-023-33893-7 (PMC10172346; doi:10.1038/s41598-023-33893-7)
Supplement: Supplementary file 1 — Supplementary Information. [file 41598_2023_33893_MOESM1_ESM.pdf]

SUPPLEMENTARY INFORMATION

Supplementary Figure

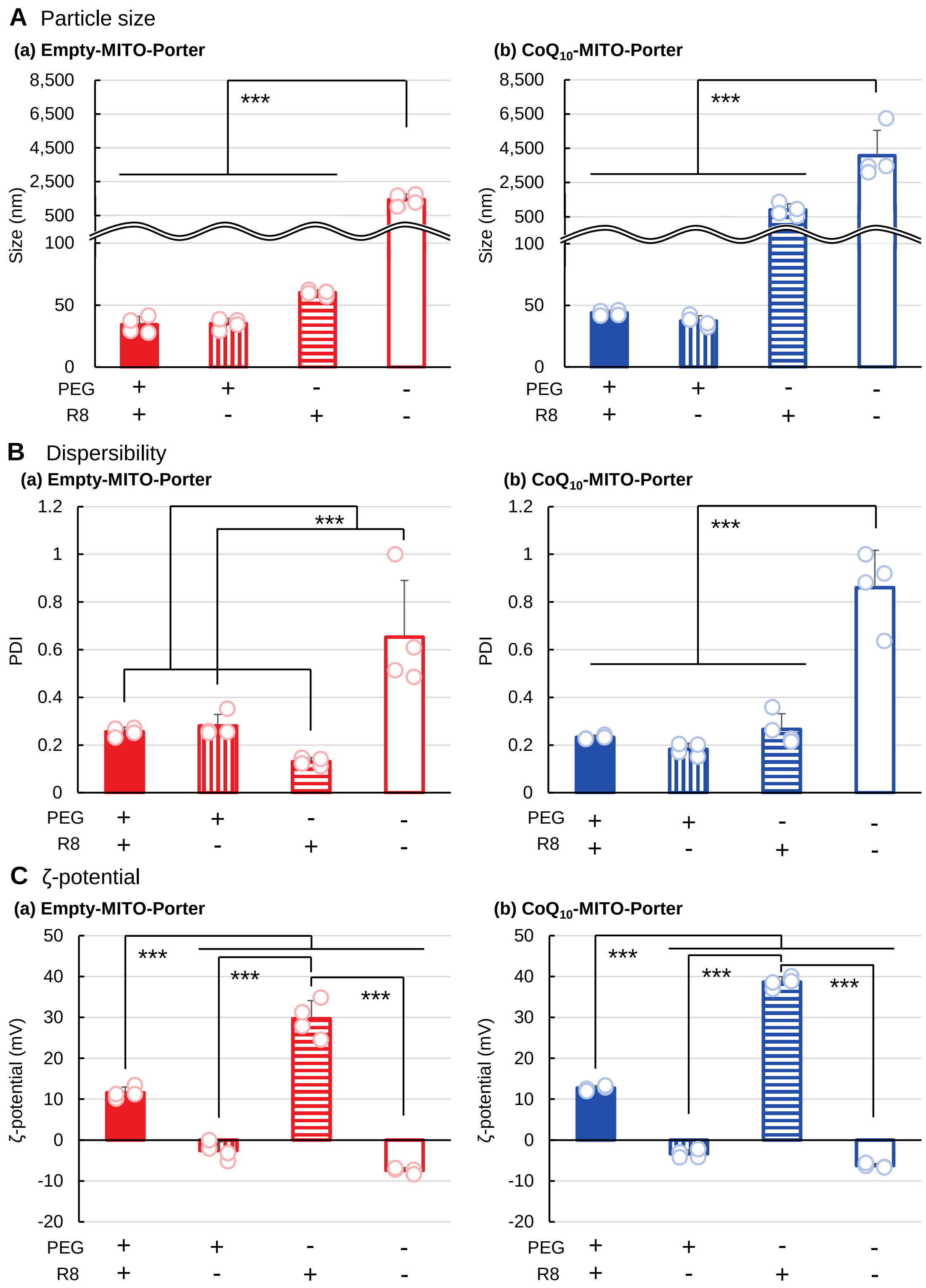

Figure S1 Physical properties of the MITO-Porter before dialysis. The physical properties of the MITO-Porter with or without PEG, R8 and CoQ_{10} after being prepared by the microfluidic device were evaluated. The prepared LNPs are characterized by three indices: (A) Particle size, (B) Dispersibility, (C) ζ-potential. The physical properties of (a) Empty-MITO-Porter and (b) CoQ_{10}-MITO-Porter are compared to consider the effect of CoQ_{10}, a poorly water-soluble molecule. Circles represent the values of 4 individual samples and bars are the mean (n=4). Data represented the mean ± S.D. (n=4). The significant differences were calculated by two-way ANOVA, followed by Tukey test (***p<0.001).

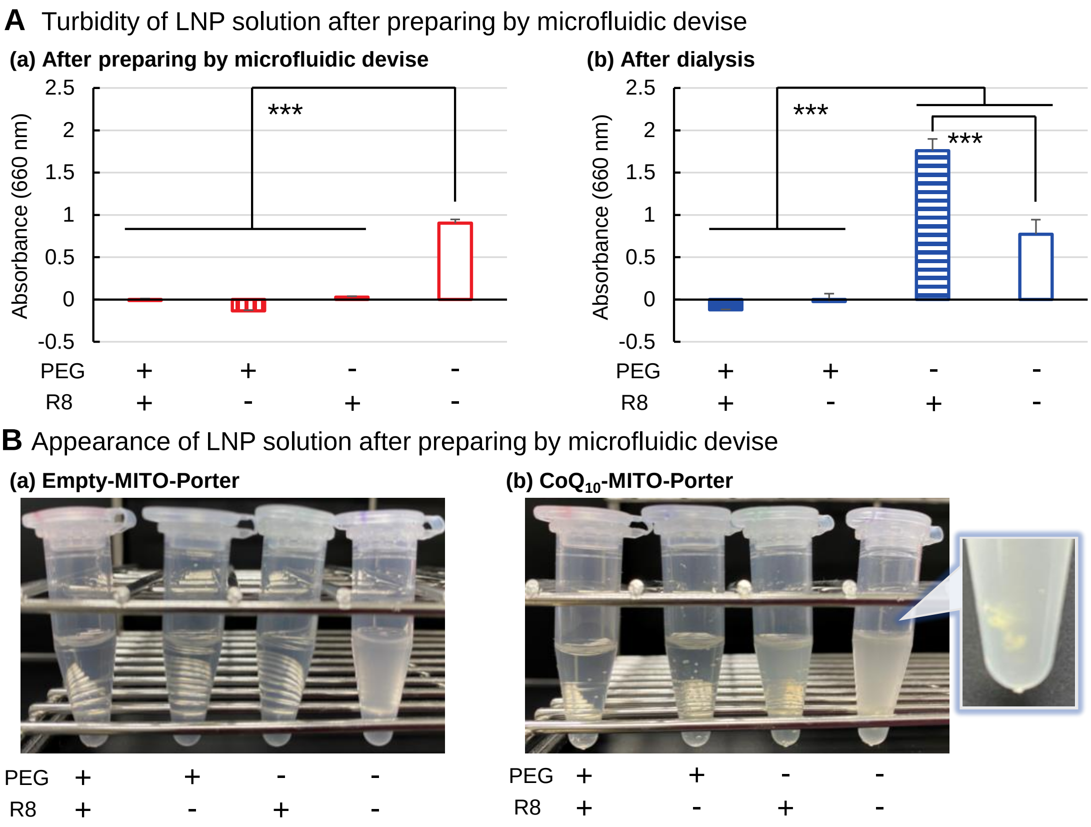

Figure S2 Appearance of a MITO-Porter solution before dialysis. The appearance of a MITO-Porter solution with or without PEG, R8 and CoQ_{10} after being prepared by a microfluidic device were evaluated. (A) Turbidity of LNP solution. Data represented the mean ± S.D. (n=4). The significant differences were calculated by two-way ANOVA, followed by Tukey test (***$p<0.001$). (B) Appearance of LNP solution. (a) Empty-MITO-Porter and (b) CoQ_{10}-MITO-Porter are compared.

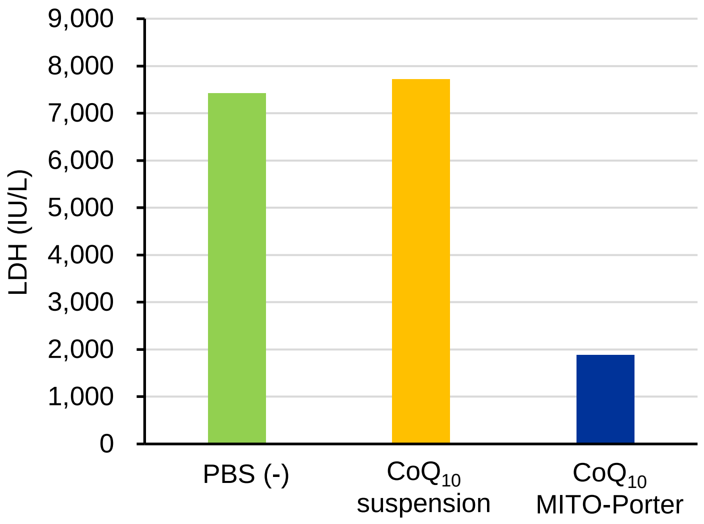

Figure. S3 Measurement of serum LDH levels. Serum lactate dehydrogenase (LDH) was evaluated, as biochemical evaluation other than serum ALT, a marker of liver function, in the APAP-induced liver injury model mice. Data represented the mean (n=2-4).

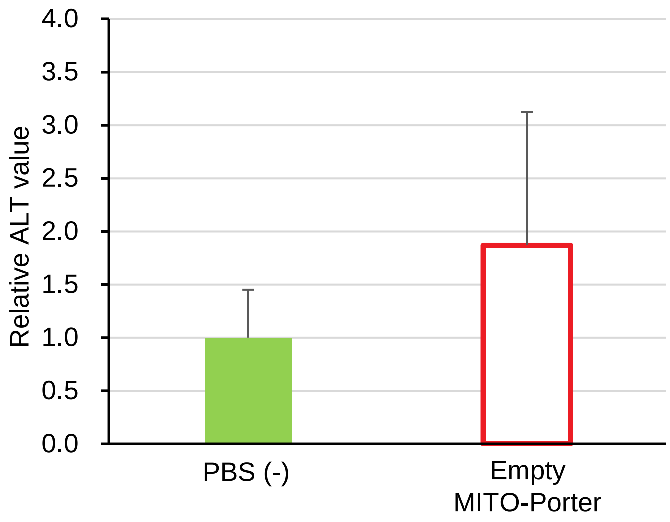

Figure. S4 The values for relative ALT. The empty-MITO-Porter was performed as a control experiment to confirm that the delivery of CoQ_{10}-MITO-Porter system protects against APAP liver injury. Data represented the mean ± S.D. (n=4). In Fig. S4, significant differences between PBS (-) group and empty-MITO-Porter group were calculated by the Unpaired t-test, and no significant difference was found. The physical properties of the administered empty-MITO-Porter are reported Table S8 in supplementary information.

Supplementary Table

Table S1 Characteristics of the MITO-Porters.

Sample	Diameter (nm)	Polydispersity index	ζ-potential (mV)
After preparing by microfluidic devise (before dialysis)			
Empty-MITO-Porter [PEG (+), R8 (+)]	34.2 ± 6.7	0.257 ± 0.0187	11.6 ± 1.4
Empty-MITO-Porter [PEG (+), R8 (-)]	35.2 ± 4.2	0.280 ± 0.0488	-2.6 ± 2.2
Empty-MITO-Porter [PEG (-), R8 (+)]	60.1 ± 2.4	0.131 ± 0.0164	29.7 ± 4.4
Empty-MITO-Porter [PEG (-), R8 (-)]	1,433.3 ± 352.0	0.657 ± 0.238	-7.4 ± 0.6
After dialysis			
Empty-MITO-Porter [PEG (+), R8 (+)]	59.3 ± 2.9	0.528 ± 0.0284	10.1 ± 0.6
Empty-MITO-Porter [PEG (+), R8 (-)]	54.9 ± 3.8	0.342 ± 0.104	-4.7 ± 0.9
Empty-MITO-Porter [PEG (-), R8 (+)]	75.4 ± 1.8	0.119 ± 0.0152	26.7 ± 1.3
Empty-MITO-Porter [PEG (-), R8 (-)]	5,077.8 ± 558.5	0.593 ± 0.260	-8.3 ± 0.5
After preparing by microfluidic devise (before dialysis)			
CoQ_{10}-MITO-Porter [PEG (+), R8 (+)]	43.9 ± 2.1	0.233 ± 0.00787	12.7 ± 0.6
CoQ_{10}-MITO-Porter [PEG (+), R8 (-)]	37.2 ± 4.4	0.183 ± 0.0255	-3.4 ± 0.9
CoQ_{10}-MITO-Porter [PEG (-), R8 (+)]	899.9 ± 363.3	0.266 ± 0.0651	38.7 ± 1.3
CoQ_{10}-MITO-Porter [PEG (-), R8 (-)]	4,075.3 ± 1,467.8	0.860 ± 0.156	-6.3 ± 0.5
After dialysis			
CoQ_{10}-MITO-Porter [PEG (+), R8 (+)]	54.4 ± 4.8	0.289 ± 0.0188	12.6 ± 1.4
CoQ_{10}-MITO-Porter	56.4 ± 3.6	0.206 ± 0.246	-4.8 ± 0.2

[PEG (+), R8 (-)]			
CoQ_{10}-MITO-Porter	387.9 ± 54.2	0.804 ± 0.119	37.6 ± 1.5
[PEG (-), R8 (+)]			
CoQ_{10}-MITO-Porter	7,447.5 ± 1,302.1	0.813 ± 0.232	-8.6 ± 0.8
[PEG (-), R8 (-)]			

Particle size, polydispersity index and ζ-potential were summarized for the empty-MITO-Porter and the CoQ_{10}-MITO-Porter after being prepared by a microfluidic device and after dialysis. Data represents the mean ± S.D. (n=4).

Table S2 Absorbance measurements of MITO-Porter suspension.

Sample	660 nm Absorbance		
After preparing by microfluidic devise (before dialysis)			
Empty-MITO-Porter [PEG (+), R8 (+)]	-0.0107	±	0.0222
Empty-MITO-Porter [PEG (+), R8 (-)]	-0.134	±	0.0092
Empty-MITO-Porter [PEG (-), R8 (+)]	0.0270	±	0.0106
Empty-MITO-Porter [PEG (-), R8 (-)]	0.902	±	0.0442
CoQ_{10}-MITO-Porter [PEG (+), R8 (+)]	-0.122	±	0.0055
CoQ_{10}-MITO-Porter [PEG (+), R8 (-)]	-0.0237	±	0.0932
CoQ_{10}-MITO-Porter [PEG (-), R8 (+)]	1.76	±	0.139
CoQ_{10}-MITO-Porter [PEG (-), R8 (-)]	0.772	±	0.171
After dialysis			
Empty-MITO-Porter [PEG (+), R8 (+)]	0.225	±	0.0071
Empty-MITO-Porter [PEG (+), R8 (-)]	0.204	±	0.0384
Empty-MITO-Porter [PEG (-), R8 (+)]	0.205	±	0.0175
Empty-MITO-Porter [PEG (-), R8 (-)]	0.783	±	0.0322
CoQ_{10}-MITO-Porter [PEG (+), R8 (+)]	0.0117	±	0.0032
CoQ_{10}-MITO-Porter [PEG (+), R8 (-)]	0.0347	±	0.0402
CoQ_{10}-MITO-Porter [PEG (-), R8 (+)]	1.10	±	0.123
CoQ_{10}-MITO-Porter [PEG (-), R8 (-)]	0.657	±	0.0407

Absorbance intensity was summarized regarding empty-MITO-Porter and CoQ_{10}-MITO-Porter after microfluidic device preparation and after dialysis. Data represented the mean ± S.D. (n=4).

Table S3 **Two-way ANOVA analysis results of the physicochemical properties of MITO-Porters prepared using a microfluidic device (before dialysis).**

Sample	Parameter	Factors	P-value by 2-way ANOVA	Significant difference
Empty-MITO-Porter	Particle size	PEG	3.4×10^{-6}	＜0.001
		R8	4.8×10^{-6}	＜0.001
		Interaction between two factors	4.9×10^{-6}	＜0.001
	Dispersibility	PEG	6.6×10^{-2}	*NS*
		R8	7.7×10^{-4}	＜0.001
		Interaction between two factors	1.5×10^{-3}	＜0.01
	ζ-potential	PEG	2.4×10^{-4}	＜0.001
		R8	1.5×10^{-10}	＜0.001
		Interaction between two factors	1.6×10^{-6}	＜0.001
CoQ_{10}-MITO-Porter	Particle size	PEG	3.1×10^{-5}	＜0.001
		R8	1.3×10^{-3}	＜0.01
		Interaction between two factors	1.2×10^{-3}	＜0.01
	Dispersibility	PEG	2.6×10^{-6}	＜0.001
		R8	3.8×10^{-5}	＜0.001
		Interaction between two factors	7.1×10^{-6}	＜0.001
	ζ-potential	PEG	6.3×10^{-12}	＜0.001
		R8	5.7×10^{-17}	＜0.001
		Interaction between two factors	4.4×10^{-13}	＜0.001

Physical property values of the MITO-Porters were subjected to a 2-way ANOVA analysis to compare the effect of 2 factors, “PEG” and “R8”. A significant interaction between the two factors was found and a simple main effect test was performed. Significant differences (*NS*: not significant, $p<0.01$, $p<0.001$) were calculated by a simple main effect test, followed by Tukey test.

Table S4 Two-way ANOVA analysis of the turbidity of a MITO-Porter solution prepared using a microfluidic device (before dialysis).

Sample	Factors	P-value by 2-way ANOVA	Significant difference
Empty-MITO-Porter	PEG	6.4×10^{-10}	＜0.001
	R8	1.8×10^{-9}	＜0.001
	Interaction between two factors	4.9×10^{-10}	＜0.001
CoQ_{10}-MITO-Porter	PEG	5.2×10^{-10}	＜0.001
	R8	4.1×10^{-5}	＜0.001
	Interaction between two factors	5.2×10^{-6}	＜0.001

Absorbance intensity of the MITO-Porters were subjected to a 2-way ANOVA analysis to compare the effect of 2 factors that are "PEG" and "R8". A significant interaction between the two factors was found and then a simple main effect test performed. Significant differences (p<0.001) are calculated by a simple main effect test, followed by Tukey test.

Table S5 **Characteristics of LNPs.**

Sample	Diameter (nm)	Polydispersity index	ζ -potential (mV)
Empty-MITO-Porter	40.8	0.371	11.2
CoQ_{10}-MITO-Porter	51.5	0.327	8.5

Particle properties of the empty-MITO-Porter and CoQ_{10}-MITO-Porter for SAXS analysis (Fig. 4B) are summarized. Number of data is 1.

Table S6 **Characteristics of the CoQ_{10}-MITO-Porter labelled with DiD.**

Sample	Diameter (nm)	Polydispersity index	ζ-potential (mV)
DiD-CoQ_{10}-MITO-Porter	52.8 ± 2.7	0.269 ± 0.03	9.5 ± 1.3

Particle properties of the CoQ_{10}-MITO-Porter labeled with DiD for pharmacokinetics analysis (Fig. 5) are summarized. Data represent the mean ± S.D. (n=3).

Table S7 **Characteristics of the CoQ_{10}-MITO-Porter.**

Sample	Diameter (nm)	Polydispersity index	ζ-potential (mV)
CoQ_{10}-MITO-Porter	52.2 ± 4.0	0.307 ± 0.05	14.0 ± 1.8

Particle properties of the CoQ_{10}-MITO-Porter for animal studies against APAP liver injury (Fig. 6) are summarized. Data represent the mean ± S.D. (n=3).

Table S8 **Characteristics of the empty-MITO-Porter.**

Sample	Diameter (nm)	Polydispersity index	ζ-potential (mV)
Empty-MITO-Porter	53.8 ± 1.7	0.473 ± 0.121	12.9 ± 1.3

Particle properties of the empty-MITO-Porter for animal studies against APAP liver injury (Fig. S4) are summarized. Data represent the mean ± S.D. (n=4).
